# Senior manager leadership competencies for quality residential aged care: an Australian industry perspective

**DOI:** 10.1186/s12913-022-07911-9

**Published:** 2022-04-14

**Authors:** Nathan Dawes, S. M. Topp

**Affiliations:** College of Public Health, Medical & Veterinary Sciences, Division of Tropical Health and Medicine, James Cook University, JCU Townsville I Douglas I Building 41 I Room 114, 1 James Cook Drive, Townsville, QLD 4811 Australia

**Keywords:** Leadership, Management, Skills, Residential aged care, Industry experts

## Abstract

**Background:**

Documented poor quality and standards of care in Australia’s residential aged care (RAC) sector have highlighted a need to better understand the role of and skills required by, RAC senior management personnel to address these concerns. This study examined which senior management leadership skills and personal qualities are necessary to deliver and strengthen the quality of RAC, with the aim of improving understanding of the professional development needs of leaders in the sector.

**Methods:**

We conducted 12 in-depth interviews with Australian aged care industry experts, including academics, and representatives from the primary health network, consumer, and provider advocate groups. Abductive, thematic analysis incorporated coding derived from existing leadership skills frameworks as well as inductively identified themes.

**Results:**

Identified leadership skills were grouped into five domains including i) workforce development and retention, ii) governance and business acumen; iii) health systems knowledge; iv) stewardship and v) responding to regulatory and political contexts. Skills particularly emphasised by participants were those required to recruit and retain a skilled workforce, manage relationships, and promote a positive organisational culture and employee wellbeing.

**Conclusions:**

RAC senior managers require a complex mix of business, human resource management, and clinical skills to deliver quality care in Australia’s complex RAC setting. The lack of any professional development framework to guide the acquisition or updating of those skills is a concern.

## Background

The global population is rapidly ageing [1]. In 2020, there were approximately 980 million individuals aged 60 years and over and by 2050 this figure is expected to reach 2.1 billion [1]. Australia is no exception, with approximately 25% of the population projected to be 65 years and over by 2057 [2]. As the proportion of Australia’s aged population increases, there has been a concurrent rise in demand for residential aged care (RAC), care that is capable of delivering high-quality services to older persons with complex co-morbidities such as multiple chronic non-communicable diseases and dementia [2]. Yet, the inadequacies of Australian RAC services made public as part of the *Royal Commission into Aged Care Quality and Safety*, demonstrated numerous incidences of neglect and substandard clinical services [3]. The same commission identified leadership skills and strategies required by managers to promote quality of care as lacking, by comparison to international RAC services and other Australian mainstream health care organisations [3].

Leadership is considered a foundation stone for improving health care quality [4] and includes the ability to identify priorities and provide strategic direction to multiple actors within the health system [5]. Effective health leadership supports and fosters a culture of continual learning and improvement; a culture that ensures care recipients are at the centre of care planning and delivery and where staff are supported to provide safe, effective and compassionate care [5]. Xing, Song and Yan (2020) also suggest the personal qualities of health care leaders are linked to increased quality through the effects they have on staff wellbeing and patient engagement [6], and the empowerment of care recipients to make informed decisions regarding their own care [7]. Leadership that promotes and enables patient engagement contributes to improving quality of care, producing better health outcomes and often reduces health care costs [7].

While leadership is conceptualised in many ways, most frameworks recognise four central characteristics [8]. Respectively, these include i) leadership is a process, ii) involves different forms of influence, iii) occurs in groups and iv) involves clear vision and a common goal [8]. In health care, a *skills perspective* (approach) to leadership is often adopted [8], with a view to strengthening the quality outcomes of an organisation by recognising the abilities required to lead quality performance [9]. While health care leadership is a broad term, with a diverse range of applications [10], the *skills perspective* can be used to identify and describe the competencies (knowledge and skills) that are required by managers to influence high-quality care across multiple health care settings, including residential aged care [1]. Here, we define a leadership competency as an expected level of performance that results from an integration of knowledge, skills, abilities and judgment and recognise such attributes as integral to influencing quality [11]. Extending this definition, leadership attitudes and behaviours can also be considered important leadership characteristics that contribute to high-quality care [12].

Although the term leadership competency is used interchangeably with other related concepts including leadership traits, capabilities and attributes, it is important to acknowledge that the definition and application of each term differ [13]. Traits are ingrained behaviours that are mostly permanent and difficult to change [14], whereas attributes are usually understood to be specific behaviours learned as part of external experiences [15]. Leadership competencies are a way of measuring how well an individual does certain things, taking into consideration knowledge, skills and attributes [16]. Competency differs from capability. Whereas capability may be defined as having the ability to do something (with improved capability arising from the improvement of skills), “competence” references the degree of skill in the task’s performance [17]. Improved capabilities may lead to competence [18].

Substantial research has been conducted around leadership skills in mainstream health care organisations [11]; yet comparatively little is known about the skills or combinations of skills required in the distinctive setting of residential aged care [18]. It is therefore important to recognise that the demands of, and thus skills required by, managers in residential aged care services may differ from other health care organisations. The continuous nature and complexity of clinical services required by RAC residents [19]; specific regulatory requirements associated with the RAC sector, and facility-level business operations that must accommodate both clinical and broader lifestyle considerations [19], all highlight the need for leadership capabilities that include (a combination of) skills distinct from those required in non-residential mainstream healthcare organisations [18]. With the demand for residential aged care services increasing in Australia and concurrent concerns regarding the quality of that care [2], there is a clear need to understand which types and combinations of skills are required by senior managers to provide effective leadership in this complex landscape.

The Australian Aged Care Leadership Capability Framework was developed by Aged and Community Services Australia [ACSA] in 2014. This framework is behaviorally based, and the capabilities are illustrated by a set of indicative behaviours appropriate to multiple levels of leadership, including frontline, middle and senior management roles [20]. The design of the framework is indicative of an intent to provide broad guidance to leadership across a range of aged care agencies, acute, community and residential aged care provider organisations [20]. However, the Australian Aged Care Leadership Capability Framework (2014) makes no specific mention of the senior manager role, nor the competencies linked to this leadership responsibility and high-quality care, beyond mention of the capability of ‘person-centred focus’. Indeed, to the author’s knowledge, no sector-specific skills or leadership competency frameworks focusing on the professional development and attainment of competencies required by RAC senior managers to promote quality of care has been produced in Australia. Moreover, few studies have explored the topic in-depth.

Recent reviews of the literature have synthesised evidence concerning the role of leadership in promoting quality of care in residential aged care, globally. In a scoping review of the literature that focused on senior management leadership to promote quality of care in residential aged care, Dawes and Topp (2019), for example, found 14 studies, the majority of which focused on leadership styles, not competencies; and the majority of which (*n* = 12) reported on RAC in the United States of America [21]. Moreover, Jeon, Merlyn, and Chenoweth (2010); and Zonneveld, Pittens, and Minkman (2021) each explored the role of leadership across multiple organisational levels, with findings that focussed on leadership behaviours connected to certain leadership styles including relationship-oriented, task-oriented and context-dependent leadership [22, 23]. Most recently, an original research study by O’Toole, Bamberry and Montague (2021) examined the perceptions of leadership by senior managers and identified the crucial requirements for successful leadership within the Australian residential aged care industry [24]. Findings clearly demonstrated recognition among senior managers that effective leadership skills are required to successfully deliver quality care and resident satisfaction (O’Toole, Bamberry and Montague, 2021) but reported leadership competencies were again considered across multiple organisational levels with none specifically linked to the senior manager role [24].

With a view to addressing the gap in current knowledge regarding the combination of skills required by residential aged care senior managers in Australia, this study aimed to qualitatively explore the views of a range of aged care industry experts regarding the senior management leadership competencies required to ensure quality of care.

## Methods

### Study design

We conducted an exploratory qualitative study to understand, from the perspective of Australian aged care industry experts, the leadership competencies required by Australian RAC senior managers to influence high-quality care. For the purpose of the current study, Australian aged care industry experts are those in a professional role that is either an aged care researcher, primary health network representative, consumer or provider advocate.

Qualitative methods support the examination of underlying reasons, opinions, and motivations of individual participants. We conducted in-depth interviews (IDIs) using probes such as ‘why’, ‘how’ and ‘what’ to gain a deeper understanding of participants’ views and experiences regarding the senior manager leadership skills that influence quality of care in Australian RAC organisations.

### Study setting

The current study was completed with representatives who contribute to or advise regarding the delivery of aged care services in Australia. Examples of different ‘levels’ of aged care include: i) entry-level community-based care at home; ii) higher levels of care at home (Home Care Packages Program), and when living at home is not an option iii) residential aged care [25]. This study focused specifically on the role of senior managers in providing quality care in the Australian residential aged care setting. Residential aged care provides health care services and accommodation for older people who are unable to continue living independently in their own homes [26].

In Australia, residential aged care providers can span a range of different sectors including religious, charitable, community, for-profit and government organisations [26]. Typical services may include accommodation, personal care assistance, clinical care and a range of social care activities including recreational activities and emotional support. Approximately 250,000 older Australians received permanent residential aged care at some time during the financial year 2019/2020 [27].

### Participant recruitment

Industry representatives [experts] engage with stakeholders across multiple levels of the Australian aged care system and inform sector-wide policy development and governance arrangements [28]. We identified three major groupings of industry representatives. First, those who work for national peak advocacy bodies that provide support, advocacy, and policy development services for their members [29]. Peak advocacy bodies provide a mechanism for collating and representing the experiences of a heterogeneous membership, on issues relating to consumer and labour protections, quality of care and aged care regulation [28].

The second group of industry experts include those working for primary health networks (PHNs) statutory bodies funded by Australia’s federal government [30]. By conducting population needs assessments and commissioning services, PHNs aim to improve health care efficiency and effectiveness (particularly for those at risk of poor health outcomes including older care recipients) and enhance the coordination of health services at local, regional and national levels [30]. PHN representatives possess the skills and knowledge to evaluate and monitor the effectiveness of health services against local population health needs, including those provided in RAC [31]. They also provide education and training aimed at developing workforce skills to positively influence quality performance and are thus well-positioned to describe the link between senior manager leadership skills and quality of care delivered by Australian aged care services [31].

The third group of experts are aged care researchers who aim to improve understanding of, and produce evidence, tools and resources to improve, health and policies and services in the aged care sector [32]. Like peak body advocates, aged care researchers tend to engage with a variety of stakeholders with a view to exploring or developing knowledge to inform national policy or implementation [28, 32] including the skills required to develop leadership training programs for managers to drive best practice at national and facility levels [10].

Purposive sampling was used to identify and select information-rich participants from the three expert groupings described earlier, with knowledge of and experience working within the Australian aged care sector. Participant selection was deliberate and aged care industry experts were recognised as possessing specific knowledge of health service needs of older persons in Australia and capable of reflecting critically on the link between senior manager leadership skills and quality RAC. Snowball sampling was employed to incorporate eligible participants who may have been missed during initial recruitment. Two additional interviewees were recruited using this technique. To be included in the study, participants were required to: i) be aged 18 years and above and ii) be either an aged care researcher, primary health network representative, consumer or provider advocate.

Using a combination of aged care industry experience and a comprehensive desk search, the first author (ND) developed a list of eligible individuals and organisations using public access contact information including Primary Health Network organisations, Department of Health government websites, national research institutes and Australian University websites. Participants were then emailed an invitation for involvement. Participants were also deemed as eligible for participation if they represented national government committees including the National Aged Care Advisory Council and Aged Care Sector Committee. Both government committees support aged care policy development and implementation.

Overall, 12 in-depth interviews were conducted by ND between December 2020 and February 2021, via video conferencing (*n* = 11) and telephone (n = 1). Interviews were conducted with provider advocates (*n* = 6), consumer advocates (*n* = 3) researchers (*n* = 2) and primary health network (PHN) representatives who are involved in commissioning Australian aged care services (n = 1) (Table 1). One participant who was an aged care Researcher also represented the National Aged Care Advisory committee, while two consumer advocates were members of the Aged Care Advisory group.

**Table 1:** Description of participants based on professional role and organisation type

Participant ID	Position	Organisation Type	Government affiliation
**1**	Chief Executive Officer (CEO)	Provider advocacy	
**2**	National Policy and Advocacy Manager	Provider advocacy	
**3**	Chair of Board – Non – Executive Director	Consumer advocacy	Aged Care Advisory group
**4**	Chief Executive Officer (CEO)	Consumer advocacy	Aged Care Advisory group
**5**	Chief Executive Officer (CEO)	Provider advocacy	
**6**	Senior Policy and Engagement Officer	Provider advocacy	
**7**	Professor – Academic	Research Institution	National Aged Care Advisory committee
**8**	Queensland State Manager	Provider advocacy	
**9**	Program Director	Research Institution	
**10**	Executive Director	Provider advocacy	
**11**	Project Manager – Aged Care	Primary Health Network	
**12**	President	Consumer advocacy	

The interview guide canvassed the role of the industry expert, their perceived link (if any) between senior managers and RAC quality of care, current and potential challenges associated with delivering high-quality RAC, and the leadership skills required to address these concerns. All participants provided written informed consent and agreed to the interview being audio-recorded and transcribed. Each participant was provided with a copy of the interview transcription and an opportunity to correct or remove data prior to the analysis.

### Data management and analysis

Abductive, thematic analysis incorporated coding derived from existing leadership skills frameworks as well as inductively identified themes. To identify major and minor themes, we took the following steps: i) handwritten memos were collated immediately after each interview to ensure that a reflexive stance was maintained in relation to the research situation, participants and documents under study; ii) familiarisation through careful and repeated reading of transcripts and research memos, noting emergent themes; iii) each individual participant was emailed a copy of the transcribed verbatim to ensure that the investigators records corresponded with those of the participants from whom those data were derived; iv) open coding in which codes were created based on identified themes, codes were assigned to specific sections of transcripts; v) data display using matrices including summary tables.

## Results

### Overview

We present findings from this qualitative analysis under five inductively identified skill domains including i) workforce development and retention, ii) governance and business acumen; iii) health systems knowledge; iv) stewardship and v) responding to regulatory and political contexts. In the following sections, these overarching domains, and the more specific leadership skills they encompass are referred to simply as ‘domains’ and ‘skills’ respectively to improve clarity. Interview participants are identified only by generic labels (as outlined in Table 1) of ‘consumer advocate’, ‘provider advocate’, ‘Primary Healthcare Network’ or ‘Researcher’.

### Workforce development and retention

Skills in this domain included a manager’s ability to develop a workforce with an appropriate balance of clinical skills across the organisation. To achieve this optimal skill mix, a manager’s ability to recruit health care personnel across key service areas, with the knowledge to service a range of complex co-morbidities and psychosocial needs specific to an older demographic, was reported by a majority of participants as critical to quality of care.

*The ability of a leader to choose, recruit and retain key people across the core health services areas is so important to delivering quality care****Consumer advocate – ID4***

Critical to being able to support recruitment and retention, five participants comprising both provider and consumer advocates, additionally noted the importance of human resource management skills; including the ability to negotiate with staff and being compassionate to an employee’s needs within and outside of the workplace.

*Human resource management is so essential to making quality health care occur.****Provider advocate - ID2***

Alongside these more technically oriented skills, a majority of participants including primary health network representatives, consumer and provider advocates, collectively highlighted the importance of a senior manager’s relational skills. Key amongst these was the ability to nurture and build relationships with staff, communication skills and building peer support networks. The ability of senior managers to develop rapport and trusting relationships with staff, for example, was described as promoting open channels of communication among interprofessional teams and thus promoting high-quality care*.*

*So, it's being personable and being able to develop that rapport with your staff so that they trust you and they feel like they can come to see you to discuss anything regarding the health care services that they are responsible for providing.****Provider advocate - ID2***

Another participant emphasised the importance of a manager’s ability to employ communication skills involving empathy and active listening techniques, as essential to creating therapeutic relationships with residents and their families and to positively influencing care quality.

*I think every person who works in aged care, whether they're a leader or not, needs to have good communication skills in order to be able to engage in a therapeutic manner with residents, and so communication skills involve imparting empathy and involve listening.****(Researcher – ID9)***

External to the organisation, a manager’s ability to build and nurture peer support networks with other RACFs, to share expertise around business models that promote quality of care, improve business knowledge, and receive peer mentorship, was also emphasised as an important leadership skill by four provider advocates:

*People should start to build collaborations across other [aged care] organizations … so that they can bring in really top-quality people.****Provider advocate – ID5***

*Make sure that you've got a good peer network around you that you reach out for that support.* ***Provider advocate – ID8***

### Governance and business acumen

The ability of senior managers to create a governance structure to delineate power and define management roles in an organisation, was linked to quality of care. Participants viewed this skill as a strategy for managers to set rules, procedures, and other informational guidelines to quality improvement. Researchers, primary health network representatives, provider and consumer advocates reported skills under this domain and linked these to quality of care.

A provider advocate emphasised that senior managers should possess the knowledge to develop an organisational structure that provides executives and managers the opportunity to make informed decisions regarding health care delivery.

*The organisational structure must be designed by managers so that they can support themselves … to free up their time to make the best decisions for their health care services****Provider advocate – ID10***

A consumer advocate emphasised the importance of senior managers possessing the skills to successfully lead the operational aspects of an organisation that are linked to service provision, such as compliance management and management of resources.

*Again, leaders need to be committed to older Australians and be able to smoothly run high level operations in order to positively influence the quality of their service****Consumer advocate and Aged Care Advisory group member – ID4***

Critical to being able to support the sustainability and quality of RAC health care services, several consumer and provider advocates additionally noted the importance of a manager’s business skills such as financial management, human resource, and people management skills, as a factor contributing to quality of care. As reported by this provider advocate:

*So, there's significant financial management, sales significant clinical skills and significant human resources skills, and people management skills that are required****Provider advocate – ID2***

The capacity of a senior manager to be strategic in planning operations was also emphasised as an important leadership skill for RAC in Australia. As described by the provider advocate below, such skills were linked to effective planning to meet challenges and identify opportunities for handling the increasingly complex political, regulatory and clinical landscape of RAC in Australia:

*I think being strategic as well. So looking at opportunities and, as you were talking about before, innovation, thinking outside the square to get the best possible care for the resident.****Provider advocate – ID6***

### Health system knowledge

Skills and strategies associated with a manager’s understanding of the health care system and clinical environment were noted by a number of study participants. External to their specific facility, interviewees linked quality performance to the ability of senior managers to recognise the variations between mainstream health care organisations and RAC service provision. Five participants, who were researchers, provider and consumer advocates reported skills under this domain.

One researcher described that the quality of RAC should be focused on maintaining an older person’s quality of life, which required a unique set of leadership skills:

*So it is important to recognize the differences between acute care where the focus is on diagnosis and treatment, and aged care, where the focus is more about quality of life. It takes very different managerial skills to effectively manage each context and those who lead these organization’s need to recognise this.****Researcher and National Aged Care Advisory Committee member– ID7***

In addition to providing oversight to the clinical aspects of RAC, study participants across all expert categories suggested that senior managers should themselves possess clinical knowledge and skills to successfully embed quality health care practices within the organisation. Clinical skills included managers’ ability to recognise effective clinical care models that address the health care needs of an older demographic and the ability to recognise clinical outcomes to care.

*I think a problem where we separate out residents needs into biomedical needs, clinical needs and social needs and accommodation needs …. We need a consistent model of care that focuses solely on caring for the individual****Researcher – ID9***

*You must have a keen eye towards resident outcomes, and I would be as broad as to say clinical quality outcomes and customer experience outcomes, all of these clinical attributes are important for a manger to possess and be aware of****Provider advocate – ID 10***

One researcher suggested that if senior managers did not possess a sound level of clinical knowledge, then residents’ needs could be missed or neglected.

*So, I think the fact that we now have a lot of leaders who don't have any healthcare background has put us in a situation where resident's clinical care needs often missed and neglected.****(Researcher National Aged Care Advisory Committee member – ID7)***

### Stewardship

‘Stewardship’ encompassed leadership skills to create a positive workplace culture through creating a physical environment that encouraged employee wellbeing; promoting team cohesiveness; and helping team members overcome negative industry perceptions.

The ability of a senior manager to create a physical environment that encouraged employee wellbeing, was linked by study participants to positive workplace culture and high-quality care. The skills to promote such a physical environment included the ability to develop a workspace that promotes employee and resident comfort, with one consumer advocate describing the links to employee job satisfaction and retention and resident quality of life:

*Coming to work at a place that is comfortable each day will only improve employee performance to delivering quality care****Consumer advocate Aged Care Advisory group member – ID4***

Leadership skills to promote team cohesiveness were also linked to increased workplace culture and organisational quality performance.

*If you have a good leader, you could be working in a positive and cohesive team even though; the situation around you feels quite dire****Provider advocate – ID1***

Additionally, and potentially specific to the Australian context, participants reported the importance of stewardship skills to overcome negative public perceptions regarding RAC (in light of negative accounts heard during the recent *Royal Commission into Aged Care Quality and Safety*). The capacity to manage such perceptions were also linked to promoting a positive organisational culture and staff retention.

*I think probably the biggest challenge is the negativity within the media for the bad cases and the lack of media interest in a good case. So, it is more difficult for them to get and retain staff because of that****Consumer advocate Aged Care Advisory group member – ID4***

### Responding to regulatory and political contexts

‘Responding to regulatory and political contexts’ included the leadership skills required by senior managers to successfully interpret and respond to Australian aged care regulatory change. Researchers, provider and consumer advocates interviewed as part of this study reported skills under this domain and linked them to quality of care.

Two provider advocates suggested that while the current aged care regulatory environment can be difficult to interpret, that senior managers need to be proactive to lead RAC regulatory compliance. This process was described as senior managers initiating partnerships between regulators and their organisation to ensure a joint approach to regulatory compliance.

*Providers do need to actually look at themselves and see how they contribute to improving the overall situation … which would suggest more of a partnership-based approach between regulator and provider rather than a compliance focused approach of seeking out and punishing wrongdoing****Provider advocate – ID2***

In addition to forming external relationships with regulatory authorities, some participants emphasised that senior managers further develop their lateral thinking skills to assist in interpreting and responding to the evolving aged care regulatory and political context. This includes the ability to recognise and interpret regulatory reform and to successfully translate this change to RAC operations in order to sustain quality health care delivery.

*So, I think those external factors really require a leader to be really adaptable, to be mobile, to be a lateral thinker and responsive to the regulatory and political surroundings, in order to be effective for health service delivery****Consumer advocate – ID12***

## Discussion

Quality of care in Australian RAC has been identified as lacking, and the Royal Commission pointed to leadership as a key area requiring improvement [19]. To date, however, evidence regarding the types and combinations of leadership skills required by senior managers who lead RAC is limited [21]. Drawing on and triangulating the perspectives of experts from provider and consumer peak advocacy bodies, Primary Health Networks and aged care researchers, this study highlighted five major domains of leadership skills likely required by Australian RAC senior managers to influence quality of care, respectively: i) workforce development and retention, ii) governance and business acumen; iii) health systems knowledge; iv) stewardship and v) responding to regulatory and political contexts. Skills particularly emphasised by participants were those required to recruit and retain a skilled workforce, manage relationships, and promote a positive organisational culture and employee wellbeing. Such skills are intuitively important and noted elsewhere to be central in mainstream healthcare leadership [33, 34]. However, the emphasis on these skills as part of the current study also reflects the specific context and a number of macro-through-micro level challenges of the Australian RAC sector, including regulatory change [35] ongoing human resourcing challenges [36], and longstanding issues with work culture and morale [37].

Findings from the current study demonstrated industry experts’ perception of a strong link between a manager’s relational skills and RAC quality. These abilities included communication techniques that enable the formation of partnerships and therapeutic relationships with care recipients, their families and other immediate caregivers. Previous studies have shown the importance of effective communication with older people as a critical aspect of care quality, with ineffective communication skills often leading to older care recipients feeling inadequate, disempowered and helpless [38]. In the current context of Australian RAC, in which documented challenges to skilled workforce retention [3, 36], ongoing regulatory reform [35] and workplace culture [37] have been recently discussed, industry experts, linked the skill of partnering with care recipients, to influencing improved clinical outcomes and increased levels of health literacy. Specific to acute health care settings, research has also demonstrated that, from a quality-of-care perspective, the ability of managers to develop proficient communication skills, including active listening techniques often increases the accessibility and appropriateness of healthcare for older individuals [39].

Participants from the current study reported that senior managers’ knowledge regarding the design and implementation of clinical care models and other innovations, was important to achieving quality RAC. Study participants further stressed that effective senior managers required clinical knowledge and skills to address the unique and diverse health care needs of older persons. Such findings are likely to at least partially reflect concerns about widespread lack of regulatory compliance and poor quality of clinical care in the Australian RAC sector, as documented in the 2021 Royal Commission [3]. Previous studies, although mostly located in mainstream health care organisations, have also described a connection between a manager’s health systems knowledge, clinical skillset and quality of care. For example, Parand (2014) & Andreasson et al. (2017) both found that effective managers who positively influence care quality possess a range of technical skills including knowledge about treatments and technologies, health care services, and the health care environment in which the service is situated [40, 41]. In addition, Australian health agencies including the Agency for Clinical Innovation, affirm that a health service manager is central to the design and implementation of innovative clinical care models in promoting quality performance [42].

An intuitive but important finding from this study was the emphasis placed by participants on managers’ skills to recruit and retain a workforce with a diverse skill-set. Karan et al. (2021) describe human resources for health as a core building block for the quality of services across multiple settings [43] and previous research has also found that investment in more diverse staff and skill-mix can result in improved quality of care, quality of life, and employee job satisfaction [44]. A balanced practitioner skill-mix and healthier organisational culture was found by Braithwaite, Herkes, Ludlow, Testa & Lampree (2017) to positively influence health care outcomes, such as reduced mortality rates and increased quality of life [45].

Although much of this empirical work to date has been specific to mainstream health care organisations, participants from the current study confirmed the importance of these leadership skills in RAC settings too, additionally reporting skills required to enhance workforce capacity and development, such as the ability to promote a positive organisational culture and safe physical environment that supports employee wellbeing and promotes job satisfaction. As with knowledge of clinical skills, the emphasis placed by participants – particularly provider advocates – on such skills is likely influenced by the current Australian context in which low pay [46], challenges to workforce recruitment and retention [46] and weak morale [47] have all been documented.

Combined, the findings highlight the complex and multi-dimensional leadership skills required by modern RAC senior managers. Balancing a need for business and human resource acumen, with clinical expertise and relational skills implies the potential need for highly targeted professional development frameworks and support packages if industry-wide strengthening in leadership is to occur into the future. This study is the first building block in that effort.

As with the majority of studies, the design of the current study is subject to limitations. Firstly, purposive sampling was used to recruit interview participants from three categories of experts (peak bodies, PHNs, and researchers), however not all participants were able to interview due to scheduling or other issues. As a result, half the study participants were provider advocates, whose primary focus is to support the viability and sustainability of aged care service providers [28]; these experts are potentially less likely to consider the resident experience and personalised health care needs. Conversely, consumer advocates play an important role in advocating for the older person and speaking on behalf of that individual in a way that best represents their interests [48]. With an intense focus on the individualised health care needs of older Australians, consumer advocates may have less understanding of the structural elements that adversely influence RAC quality, and the leadership competencies required. Finally, although 31 Australian PHNs represent jurisdictions defined by geographical scope, our study only included PHN representatives from North Queensland and thus likely reflected some concerns specific to that region.

While acknowledging these limitations of representation and standpoint, the use of purposive sampling was deliberately used to ensure representation of all types of expertise in some form. Close attention was paid to the positionality of each expert during analysis which included systematic triangulation of data from different experts. Further work will be important to broaden and deepen understanding of the field, nonetheless, the current findings constitute an important contribution to the field by delivering a starting point for mapping key leadership skills required by Australian RAC senior managers.

## Conclusion

With the demand for residential aged care in Australia increasing and concurrent concerns regarding the quality of that care, a better understanding of the leadership skills required to optimise quality performance is urgently required. The lack of any professional development framework to guide acquisition or updating of those skills is a concern; and overall, there remains a poorly defined link between quality of care and leadership in the context of Australian RAC. This study aimed to reduce this evidence gap and examine the senior management leadership skills necessary to deliver and strengthen the quality of RAC. Findings demonstrated that aged care industry experts view a range of technical, relational and administrative skills as critical to ensuring service quality. However, with ongoing concerns and challenges to RAC quality of care, more work is needed to prepare senior management personnel with the appropriate skills to positively lead quality care within Australia’s evolving RAC setting.

## Data Availability

The datasets used and analysed during the current study are available from the corresponding author on reasonable request.
